# A novel discovery of a long terminal repeat retrotransposon-induced hybrid weakness in rice

**DOI:** 10.1093/jxb/ery442

**Published:** 2018-12-20

**Authors:** Sadia Nadir, Wei Li, Qian Zhu, Sehroon Khan, Xiao-Ling Zhang, Hui Zhang, Zhen-Fei Wei, Meng-Ting Li, Li Zhou, Cheng-Yun Li, Li-Juan Chen, Dong-Sun Lee

**Affiliations:** 1 Rice Research Institute, Yunnan Agriculture University, Kunming, Yunnan, China; 2 Department of Chemistry, University of Science and Technology, Bannu, KPK, Pakistan; 3 Centre for Mountain Ecosystem Studies, Kunming Institute of Botany, Chinese Academy of Sciences, Kunming, Yunnan, China; 4 World Agroforestry Centre, East and Central Asia, Kunming, Yunnan, China; 5 Agricultural College of Kunming University, Kunming, Yunnan, China; 6 College of Agronomy and Biotechnology, Yunnan Agriculture University, Kunming, Yunnan, China; 7 Maize Research Institute, Shanxi Academy of Agriculture Sciences, Xinzhou, Shanxi, China; 8 State Key Laboratory for Conservation and Utilization of Bio-Resources in Yunnan, Yunnan Agricultural University, Kunming, Yunnan, China

**Keywords:** F_1_ hybrids, genome re-sequencing, gene expression profiles, hybrid weakness, *japonica*, LTR retrotransposon, polymorphism, rice (*Oryza sativa*)

## Abstract

Hybrid weakness is a post-zygotic hybridization barrier frequently observed in plants, including rice. In this study, we describe the genomic variation among three temperate *japonica* rice (*Oryza sativa* ssp. *japonica*) varieties ‘Aranghyangchalbyeo’ (‘CH7’), *‘*Sanghaehyangheolua’ (‘CH8’) and *‘*Shinseonchalbyeo’ (‘CH9’), carrying different hybrid weakness genes. The reciprocal progeny obtained from crossing any two varieties displayed characteristic hybrid weakness traits. We mapped and cloned a new locus, *Hwc3* (hybrid weakness 3), on chromosome 4. Sequence analysis identified that a long terminal repeat (LTR) retrotransposon was inserted into the promoter region of the *Hwc3* gene in ‘CH7’. A 4-kb DNA fragment from ‘CH7’ containing the *Hwc3* gene with the inserted LTR retrotransposon was able to induce hybrid weakness in hybrids with ‘CH8’ plants carrying the *Hwc1* gene by genetic complementation. We investigated the differential gene expression profile of F_1_ plants exhibiting hybrid weakness and detected that the genes associated with energy metabolism were significantly down-regulated compared with the parents. Based on our results, we propose that LTR retrotransposons could be a potential cause of hybrid weakness in intrasubspecific hybrids in *japonica* rice. Understanding the molecular mechanisms underlying intrasubspecific hybrid weakness is important for increasing our knowledge on reproductive isolation and could have significant implications for rice improvement and hybrid breeding.

## Teaser

Gene activation caused by long terminal repeat retrotransposon insertion in the promoter of a newly discovered gene, *Hwc3*, leads to hybrid weakness in intrasubspecific *japonica* rice hybrids.

## Introduction

Increased heterozygosity through wide hybridizations is generally favored in crops for superior qualities of traits and wider adaptability. Hybridization between diverse genomes generally generates an F_1_ generation with greater biomass and higher yield—a phenomena termed hybrid vigor or heterosis. However, in some cases hybrids between different species show defective development in terms of viability, fertility, and other phenotypic traits. Such defective hybrid characteristics are collectively called hybrid incompatibility and can be further subdivided into hybrid sterility, hybrid inviability, hybrid weakness, and hybrid breakdown, depending on the hybrid phenotype and underlying molecular mechanisms (Coyne and Orr, 2004; Bomblies and Weigel, 2007; Rieseberg and Willis, 2007; Koide *et al.*, 2008; Chen *et al.*, 2013; Yu *et al.*, 2018). These hybrid incompatibilities are the most important reproductive barriers for promoting the process of speciation (Johnson, 2008). Hybrid incompatibilities usually arise due to deleterious interactions between genes, chromosomal inversions, gene duplication, and transposons (Lynch and Force, 2000; Masly *et al.*, 2006; Kirkpatrick and Barton, 2006). Transposons are DNA fragments that have the ability to move from one position in the genome to another, causing mutations, and are divided into two categories (Wessler, 2006; Rebollo *et al.*, 2012), retrotransposons and DNA transposons. Retrotransposons are DNA fragments capable of copying themselves via an RNA intermediate, with the copy then moving into a new genomic position (Wessler, 2006). Retrotransposons are divided into long terminal repeat (LTR) and non-LTR retrotransposons, based on the presence of an LTR in their structure. Retrotransposons have been suggested to be involved in hybrid seed failure in interspecific *Arabidopsis* hybrids (Josefsson *et al.*, 2006). However, unlike the well-characterized roles of transposons in inducing hybrid incompatibility in the model organism *Drosophila* (Masly *et al.*, 2006; Chambeyron *et al.*, 2008), the involvement of transposons in causing hybrid incompatibility in plants is not yet well established.

Hybrid weakness, a form of hybrid incompatibility that appears at the post-embryonic stage during plant development, is frequently observed throughout plant taxa (Bomblies and Weigel, 2007). Hybrid weakness manifests itself through characteristic dwarfing of the F_1_ plants, chlorotic phenotype, stunted growth, necrotic tissues, defective root development, and partial or complete sterility (Bomblies and Weigel, 2007; Hannah *et al.*, 2007; Hatano *et al.*, 2012). Expression of hybrid weakness can lead to significant decreases in yield and even lethality and is thus a highly undesirable agronomic trait. Hybrid weakness in Asian rice (*Oryza sativa* L.) was first described in the 1950s (Oka, 1957). Subsequently, many genetic sources of hybrid weakness have been reported in rice crosses (Amemiya and Akemine, 1963; Chu and Oka, 1972; Okuno and Fukuoka, 1999; Ichitani *et al*., 2007, 2011; Fu *et al.*, 2013; Chen *et al.*, 2013, 2014). Hybrid weakness in monocots, especially in rice, is frequently reported (Koide *et al.*, 2008). However, the detailed mechanisms of hybrid weakness in rice are not yet fully understood. Few genes have been narrowed down to sufficiently small genomic intervals (Ichitani *et al.*, 2007, 2011; Kuboyama *et al.*, 2009). Two loci, *Hwi1* and *Hwi2*, consisting of three genes that cause hybrid weakness in an interspecific F_1_ hybrid between *O. sativa* and a wild rice *Oryza rufipogon* have been identified and cloned (Chen *et al.*, 2013, 2014). *Hwi1* consists of two genes encoding proteins with leucine-rich repeats that are receptor-like kinases, while *Hwi2* encodes a secreted putative subtilisin-like protease. The F_1_ hybrids appeared to have greater resistance response at higher temperatures (Chen *et al.*, 2014).

In addition to interspecific hybrid weakness, intrasubspecific hybrids may also show some degree of hybrid weakness, which is phenotypically similar to that observed in interspecific hybrids (Zhang, 2012; Fu *et al.*, 2013). Early studies suggested that *Hwc1* and *Hwc2* were two dominant complementary genes that, when occurring together in a hybrid background, conferred hybrid weakness (Amemiya and Akemine, 1963; Ichitani *et al.*, 2007). The *Hwc2* gene is found to be prevalent among temperate *japonica* rice cultivars but not among tropical *japonica* or *indica* varieties (Ichitani *et al.*, 2001). The *Hwc1* gene is rare and is reported to be present only in the Jamaica cultivar (Amemiya and Akemine, 1963; Ichitani *et al.*, 2001). Kuboyama *et al.* (2009) performed high-resolution mapping of *Hwc2*, narrowing the area of interest down to 19 kb, and identified five cDNAs in this region on the long arm of chromosome 4. Ichitani *et al.* (2007) performed linkage analysis and fine mapped *Hwc1* to a 60 kb region on the long arm of chromosome 1.

This study endeavored to provide a comprehensive genetic and functional analysis of hybrid weakness in rice. We describe an intrasubspecific hybrid weakness in F_1_ plants derived from the *japonica* rice varieties ‘Aranghyangchalbyeo’ (‘CH7’), ‘Sanghaehyangheolua’ (‘CH8’), and ‘Shinseonchalbyeo’ (‘CH9’) carrying different hybrid weakness genes (Wei, 2013). We observed that F_1_ hybrids produced from reciprocal crossing between any two of these plants exhibited the hybrid weakness phenotype. The plant material studied showed significant genomic variation. In our previous studies, we had reported that ‘CH8’ contained the rare hybrid weakness gene *Hwc1* (Zhang, 2012). In the present study, we identified and cloned a new locus, *Hwc3*, in ‘CH7’ that induced the expression of hybrid weakness in *japonica* rice hybrids. We suggest that the intrasubspecific hybrid weakness observed in the reciprocal hybrids in our study might be due to the interaction of three genes, *Hwc1*, *Hwc2*, and *Hwc3*. Taken together, our results suggest the possible involvement of retrotransposons in the expression of hybrid weakness in *japonica* rice hybrids. These studies will not only improve our understanding of reproductive isolation but will also assist in developing new strategies for crop improvement.

## Materials and methods

### Plant material

Three temperate *japonica* rice (*O. sativa* ssp. *japonica*) varieties ‘Aranghyangchalbyeo’ (‘CH7’, white pericarp, aromatic, and waxy type), ‘Sanghaehyangheolua’ (‘CH8’, purple pericarp, aromatic, and waxy type) and ‘Shinseonchalbyeo’ (‘CH9’, white pericarp and waxy type) carrying different hybrid weakness genes were used in this study. ‘LiyuB’, a wild-type *japonica* rice, was used as the control plant. Reciprocal crosses among ‘CH7’, ‘CH8’ and ‘CH9’ were performed to generate six F_1_ hybrid genotypes (‘CH7/CH8’, ‘CH8/CH7’, ‘CH7/CH9’, ‘CH9/CH7’, ‘CH8/CH9’, and ‘CH9/CH8’).

### Phenotypic characterization of hybrid weakness

In order to characterize the phenotype of hybrid weakness, quantitative analysis of important traits, namely plant height, root length, panicle length, and panicle number per plant, was conducted using 10 mature replicate plants of each of ‘CH7’, ‘CH8’, ‘CH9’, and their eight F_1_ hybrids. Plant height of seedlings was recorded at 3-d intervals starting from 3 d after transplantation to 60 d after transplantation. Plant height was measured as the distance from the basal nodes to the uppermost tips of the leaf blades and/or panicles. For root length measurements, 10 mature plants from each line at the 3-leaf stage were removed from the soil and the root length was measured (cm) for a period of 7 d. Panicle length was measured as the average value from the panicle neck to the panicle tip based on an evaluation of three panicles from each of 10 mature plants from each line at the maturity stage. To check the significance of results, the data were analysed by employing Prism 6.0 using one-way-ANOVA.

### Preparation of binary constructs and rice transformation

To prepare the constructs for complementation tests, a 2-kb fragment, designated AT70 and consisting of the LTR retrotransposon, and a 4-kb fragment, designated AT71 and consisting of the LTR retrotransposon and the *Hwc3* gene promoter and gene, were amplified using primers *Hwc3a* F/R and *Hwc3*b F/R (Supplementary Table S1 at *JXB* online). PCR was performed with an initial denaturation step of 3 min at 95 °C, with two PCR cycles (1 min at 94 °C, 1 min at 55 °C, and 4 min at 72 °C) followed by 33 PCR cycles (1 min at 94 °C, 1 min at 62 °C, and 4 min at 72 °C) and a final extension step at 72 °C for 10 min. The amplified products were separated by electrophoresis on a 0.8% (w/v) agarose gel stained with ethidium bromide. The amplified fragments were cloned into the HPE-203 binary vector to generate binary constructs. These constructs were transformed into *Agrobacterium tumefaciens* strain EHA105 and were used for transformation of ‘CH8’ embryogenic callus, with transgenic plants being generated.

### RNA extraction and RT-PCR analysis

To confirm the presence of the *Hwc3* candidate gene and to examine its expression pattern, RT-PCR analysis was performed on samples from the parental lines and F_1_ plants. Fresh leaf samples at tillering stage were taken from the plants and the total RNA was extracted using the TRNzol reagent (TRNzol, TianGen Biotech Co. Ltd, Beijing, China). The cDNAs were synthesized (RevertAid First Strand cDNA Synthesis Kit, Thermo Fisher Scientific, MA, USA) from 2 mg total RNA, according to the manufacturer’s protocol. The cDNA primers for the genes were designed using Primer-BLAST software (https://www.ncbi.nlm.nih.gov/tools/primer-blast/). The primers for RT-PCR are listed in Supplementary Table S1. RT-PCR was performed with the initial denaturation step of 3 min at 95 °C, followed by 30 RT-PCR cycles (20 s at 94 °C, 20 s at 58 °C, and 20 s at 72 °C) and a final extension step at 72 °C for 7 min. The amplified products were separated on 1% (w/v) agarose gel stained with ethidium bromide. The rice actin gene (*ACT1*) was used as an internal control to normalize the expression of the tested genes. Gel imaging and documentation was performed using the Gel Doc™ XR molecular imager (Bio-Rad, CA, USA).

### Bioinformatics analysis and characterization of the *Hwc3* candidate gene

The *Hwc3* gene was annotated according to the full-length cDNA sequence on the NCBI database. Sequence similarity was analysed using the BLAST program (http://www.ncbi.nlm.nih.Gov/ BLAST/) provided by the NCBI website. The promoter sequences were analysed through the Plant CARE database (http: bioinformatics. psb.ugent.be/webtools/plant care/html/). Protein homology was performed using BLASTp databases and a phylogenetic tree was constructed with ClustalW software (https://www.ebi.ac.uk/Tools/msa/clustalo/) using the neighbor-joining method.

### Whole genome re-sequencing

Total genomic DNA was extracted from a leaf sample following a modified CTAB method (Doyle and Doyle, 1990). DNA degradation and contamination were monitored on 1% agarose gels. DNA purity was checked using the NanoPhotometer® spectrophotometer (Implen, CA, USA). DNA concentration was measured using the Qubit® DNA Assay Kit in a Qubit® 2.0 Fluorometer (Life Technologies, CA, USA). A total amount of 1.5 µg gDNA per sample was used for library preparation. Libraries were generated using the TruSeq Nano DNA HT Sample Preparation Kit (Illumina, CA, USA) following the manufacturer’s recommendations. Briefly, the DNA sample was fragmented by sonication to a size of 350 bp, then the DNA fragments were end-polished using T4 DNA polymerase, A-tailed, and ligated with the full-length adapter (Illumina, CA, USA) for Illumina sequencing with further PCR amplification. Finally, PCR products were purified and libraries were analysed for size distribution using an Agilent 2100 Bioanalyzer (Agilent Technologies, CA, USA) and quantified using real-time PCR. The genomic re-sequencing was conducted by CapitalBio Technology (Beijing, China) to generate filtered paired-end 100-bp-long reads using the Illumina HiSeq 2000 platform. The raw data were processed using FastQC software (http://www.bioinformatics.babraham.ac.uk/projects/fastqc/). The filtered paired-end reads were mapped to the *Oryza sativa* ‘Nipponbare’ reference genome using the Burrows–Wheeler alignment (BWA) tool with default parameters (Li and Durbin, 2009). The aligned reads in the SAM (Sequence Alignment/Map) file were sorted using SortSam (Picard tools v1.118, http://broadinstitute.github.io/picard/), and the sorted SAM file was converted to a BAM file (binary version of SAM file) for variant calling using VarScan software (http://varscan.sourceforge.net). Single nucleotide polymorphisms (SNPs) and insertions/deletions (InDels) (<50 bp) were calculated and identified with snpEff-4.1 software (Cingolani *et al.*, 2012). Re-sequencing data have been submitted to the SRA repository. The SRA accession number is PRJNA488896.

### Microarray analysis

Three biological replicates (each consisting of total RNA from an independent plant) for each line extracted at the tillering stage were selected for microarray analysis. Total RNA extraction, cDNA and labelled cRNA synthesis and Affymetrix expression GeneChip hybridization were conducted by CapitalBio Technology. Arrays were scanned on an Affymetrix GeneChip scanner 3000 (Thermo Fisher Scientific). Data were normalized using the Robust Multi-Array Average (RMA) algorithm, which included quantile normalization and background subtraction. Differentially expressed genes were identified using SAM, at the reliability threshold (*q*-value)≤5% combined with standard-fold difference for gene screening with a fold-change≥2 (up-regulation) or ≤−2.0 (down-regulation). Gene description was recorded from NCBI BLAST (https://www.ncbi.nlm.nih.gov/) and Uniprot (http://www.uniprot.org/). The microarray records have been submitted to the NCBI database (https://www.ncbi.nlm.nih.gov/geo/query/acc.cgi?acc=GSE119431) and assigned GEO accession numbers as GSE119431.

## Results

### Characterization and inheritance of hybrid weakness in *japonica* rice hybrids

**Fig. 1.**
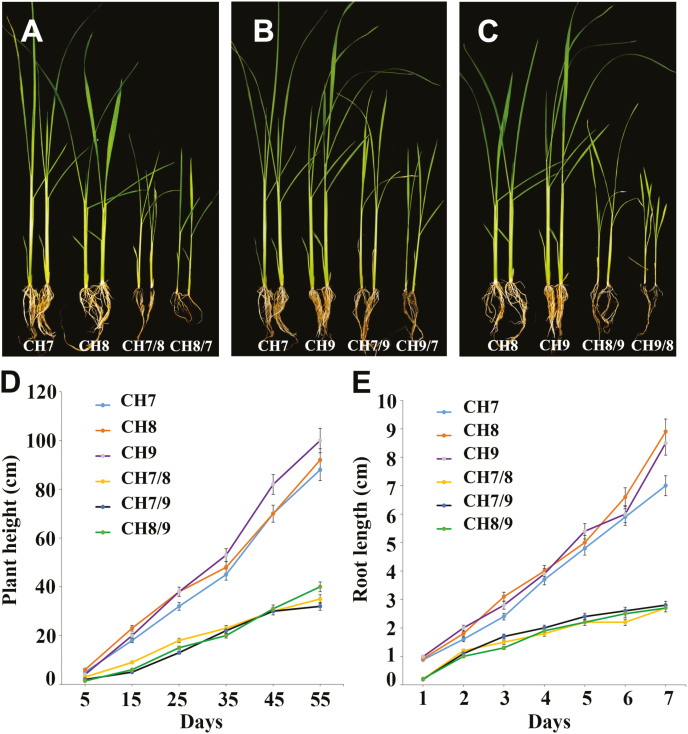
Morphological and phenotypic characterization of hybrid weakness. (A–C) Morphological evaluation of hybrid weakness in ‘CH7’, ‘CH8’, and the reciprocal hybrid ‘CH7/8’ and ‘CH8/7’ F_1_ hybrid plants (A), ‘CH7’, ‘CH9’, and the reciprocal hybrid ‘CH7/9’ and ‘CH9/7’ F_1_ hybrid plants (B), ‘CH8’, ‘CH9’, and the reciprocal hybrid ‘CH8/9’ and ‘CH9/8’ F_1_ hybrid plants (C) at 30 d after transplantation. (D) Plant height comparison of the parental plants and their hybrid weakness F_1_ progeny at the seedling and tillering stages. (E) Root length comparison of the parental plants and their F_1_ hybrids with hybrid weakness at the seedling stage. Data points in (D, E) represent the mean ±SE. (This figure is available in color at *JXB* online.)

**Fig. 2.**
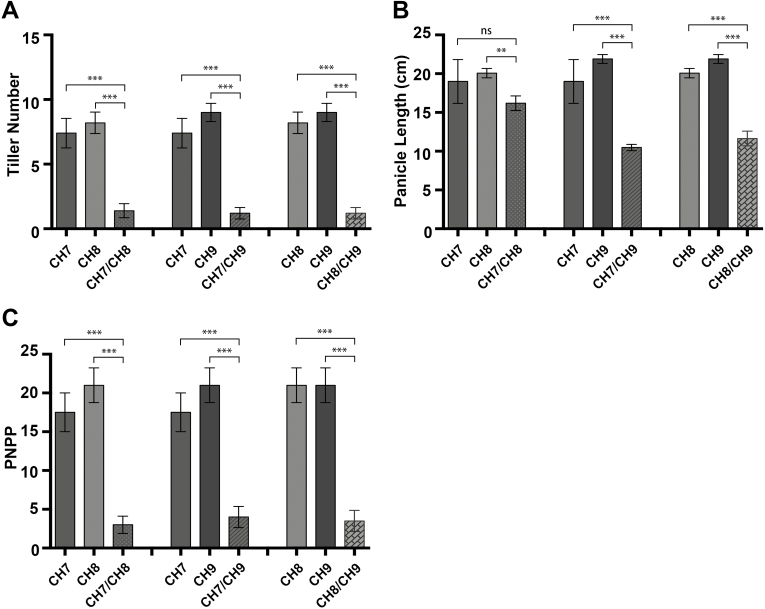
Comparison of yield components in the parents and their F_1_ progeny exhibiting hybrid weakness at the maturity stage. Comparison of tiller number (A), panicle length (B) and panicle number per plant (PNPP) (C) between parents and their F_1_ exhibiting hybrid weakness. Significant difference determined by the one-way-ANOVA: **P<0.01, ***P<0.001; ns, *P*>0.05).

F_1_ progeny obtained from all the reciprocal crosses were inferior compared with their parents with respect to all traits (Fig. 1A–C). The hybrid weakness symptoms started to appear 5 d after transplantation and became more obvious at the tillering stage. Morphological analysis showed that hybrid plants were weak, pale yellow (chlorotic), of short stature, and with wilted leaves. The overall growth of the F_1_ plants was very slow. At the seedling stage, seedling height in all the studied hybrids was significantly less than that of the parents (Fig. 1D). Compared with the parents, root development in the hybrid plants was very severely affected in terms of root number as well as morphology, and was very clearly weaker than that of the corresponding parents in all the studied hybrids (Fig. 1E). The majority of the plants that developed hybrid weakness symptoms died before reaching the adult stage, while those plants that did reach maturity produced few tillers. Important agronomic traits, namely tiller number, panicle number, and panicle length in the F_1_ plants exhibiting hybrid weakness, were significantly reduced compared with their parent plants (Fig. 2).

### Genome re-sequencing and variant discovery

Because the three *japonica* rice varieties used in this study were obtained from different geographic regions, it might be expected that the genetic differences among them would be considerable. To test this, we generated a catalog of sequence variants using whole-genome Illumina next-generation sequencing data. The raw data were filtered and mapped to the *O. sativa* ‘Nipponbare’ reference genome (Supplementary Table S2). In some parallel experiments, the three hybrid weakness plants ‘CH7’, ‘CH8’, and ‘CH9’ were crossed with *japonica* wild-type rice ‘LiyuB’. The results (Zhang, 2012) showed no hybrid weakness in the F_1_ plants, which indicated that ‘LiyuB’ does not possess a hybrid weakness gene. Therefore, ‘LiyuB’ was used as a positive control plant. Sequencing results showed that ‘CH7’, ‘CH8’, ‘CH9’, and ‘LiyuB’ produced 123792816, 142658002, 124915310, and 131997874 million reads, respectively, with a mean depth coverage of 30×. Furthermore, in ‘CH7’, ‘CH8’, ‘CH9’, and ‘LiyuB’, 93.82%, 94.06%, 93.88%, and 93.98%, respectively, of the total bases produced high-quality scores (Q30). The genetic variation among the three *japonica* rice parents, ‘CH7’, ‘CH8’, and ‘CH9’, was individually identified using Samtools. We identified a total of 574 551 polymorphic sites including 487 964 SNPs and 86 587 InDels in ‘CH7’, a total of 1 026 428 polymorphic sites including 874 667 SNPs and 151 761 InDels in ‘CH8’, and a total of 792 465 polymorphic sites including 685 118 SNPs and 10 747 InDels in ‘CH9’. These findings revealed a remarkably high density of molecular polymorphisms among each of the three studied lines. According to their position with respect to genes, SNPs and InDels were divided into different types such as upstream, downstream, intergenic, intron, and exon. The results also indicated that the distribution of SNPs and InDels was mainly in the downstream, exon, intron, intergenic, splice_site_acceptor, splice_site_donor, splice_site_region, transcript, upstream, UTR_3_prime and UTR_5_prime locations (Supplementary Tables S3, S4), and their proportions were very similar in all three parental materials (Supplementary Fig. S1A, B). These regions of the genes are mainly involved in the regulation of gene expression and we speculate that these changes might be involved in changes in gene expression profiles.

### Physical linkage map at the *Hwc3* locus and prediction of the *Hwc3* candidate gene

**Fig. 3.**
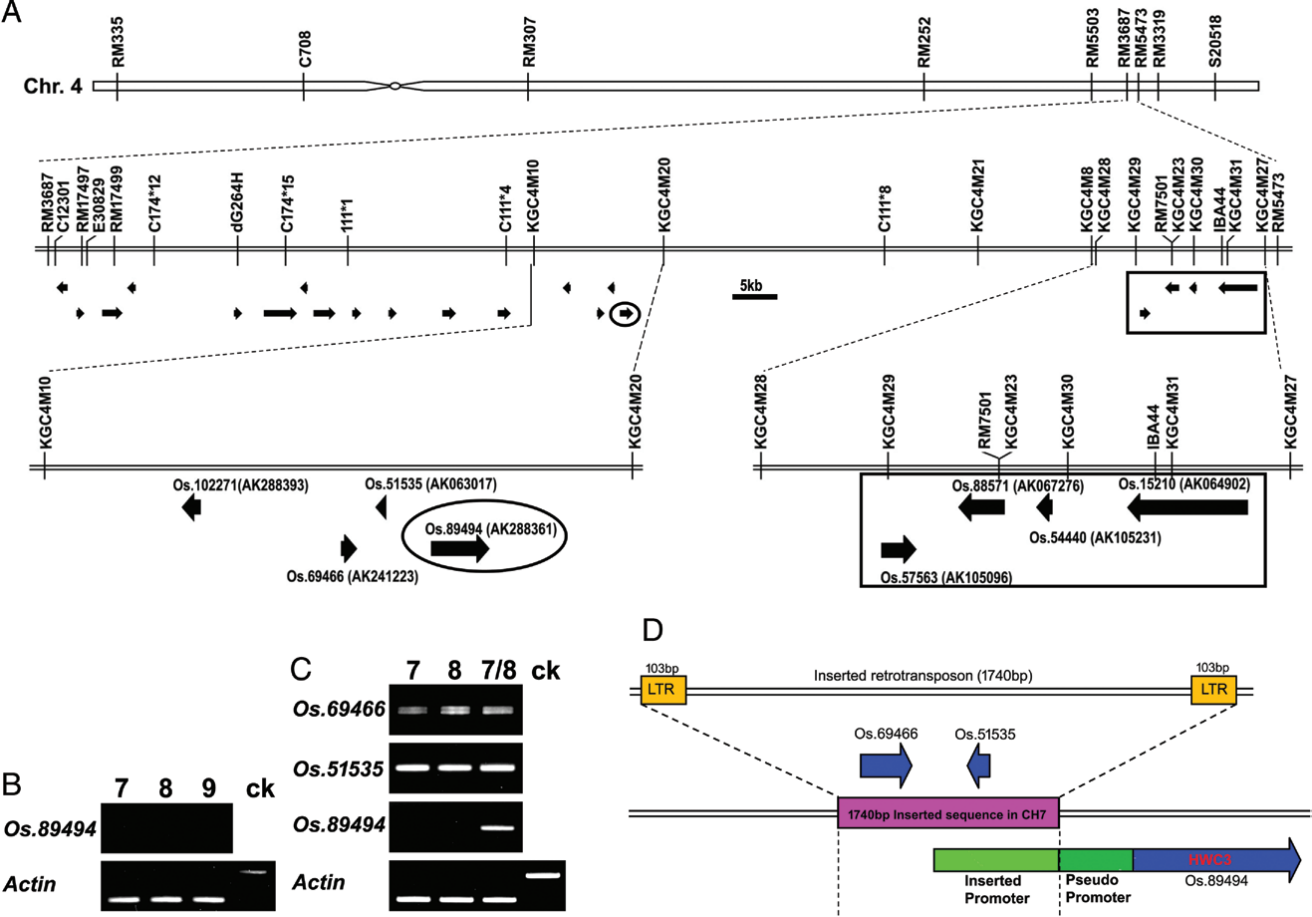
Genetic characterization of hybrid weakness. (A) Physical mapping of *Hwc3* on rice chromosome 4. Approximate location of the RM3687 and RM5473 DNA markers used for recombinants around the *Hwc*2 locus. The square denotes the *Hwc2* locus reported by Kuboyama *et al.* (2009). The circle represents the candidate *Hwc3* gene *Os.89494*. (B) RT-PCR analysis of the candidate *Hwc3* gene, where 7 represents the cDNA of ‘CH7’, 8 represents the cDNA of ‘CH8’, 7/8 represents the cDNA of ‘CH7/8’ F_1_ hybrid and ck represents the genomic DNA of ‘CH7’. The *Actin* gene was used as the internal control to normalize gene expression. (C) Gene expression analysis at the *Hwc3* locus, where 7 represents the cDNA of ‘CH7’, 8 represents the cDNA of ‘CH8’, 7/8 represents the cDNA of ‘CH7/8’ F_1_ hybrid and ck represents genomic DNA of ‘CH7’. (D) Genomic organization at *Hwc3* locus in ‘CH7’ and ‘CH8’. (This figure is available in color at *JXB* online.)

In this study, a physical linkage map was developed for the locus, based on the previous mapping and sequencing results (Yang, 2005; Wei, 2013). A sequence-tagged site (STS) marker C11112 and a cleaved amplified polymorphic sequence (CAPS) marker C1016 were used for fine mapping around the *Hwc3* locus. The *Hwc3* locus was located between the two DNA markers, RM3687 and RM5473 (Fig. 3A). The target region was narrowed down to 14 kb, and four candidate genes were identified between the markers KGC4M10 and KGC4M20, approximately 53 kb apart from *Hwc2*, as previously reported by Kuboyama *et al.* (2009) (Fig. 3A). In order to predict the candidate genes for *Hwc3*, expression analysis of the genes was studied by RT-PCR. The results indicated that only gene *Os.89494* was not expressed in ‘CH7’, ‘CH8’, and ‘CH9’ (Fig. 3B). For further molecular studies of hybrid weakness, we confined our research to ‘CH7’, ‘CH8’, and their F_1_ hybrid. Sequence analysis of this mapped region in ‘CH7’ and ‘CH8’ was performed. The results revealed that the mapped region showed significant variation between ‘CH7’ and ‘CH8’. The sequence analysis of ‘CH7’ at the *Hwc3* locus showed that an approximately 1775 bp region was deleted in the ‘CH8’ genome (Supplementary Fig. S2). A 1740 bp retrotransposon flanked at its ends by a 103 bp LTR was inserted into the promoter part of gene *Os.89494* in ‘CH7’ (Fig. 3D), while this insertion was not detected in ‘CH8’. A BLAST search showed that this sequence was found only in the temperate *japonica* rice varieties and was mostly absent from tropical *japonica* and *indica* rice varieties. Since transposon insertions are known to affect transcriptional regulation, the insertion of a LTR retrotransposon may affect the expression of adjacent genes. In order to verify the role of this gene (*Os.89494*) and the detected polymorphism at the *Hwc3* locus in inducing hybrid weakness, we analysed the gene expression pattern of the mapped genes at the *Hwc3* locus. Results showed that the two genes, *Os.69469* and *Os.51535*, showed expression in ‘CH7’ and ‘CH8’, as well as in the F_1_ hybrid (Fig. 3C). Strikingly, the gene *Os.89494* was expressed in the F_1_ hybrid, which showed hybrid weakness, but not in either of the parents (Fig. 3C). Since the parental line that did not show gene expression grew and developed normally, our results suggested that hybrid weakness in the F_1_ hybrid might be induced by the expression of this gene. Hence, we speculated that *Os.89494* could be the candidate gene for the *Hwc3* locus.

### Complementation test

**Fig. 4.**
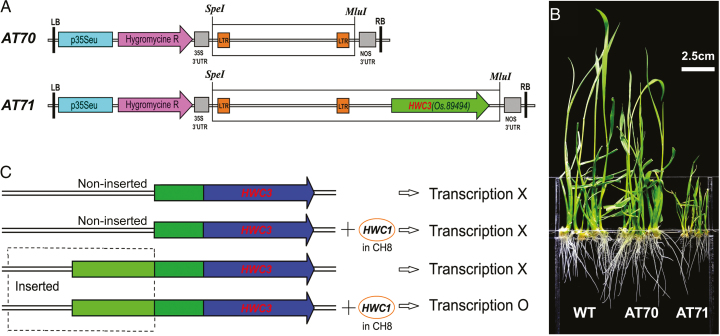
Genetic complementation and postulated model of hybrid weakness. (A) AT70 and AT71 segments derived from ‘CH7’ used for complementation tests. (B) Independent transformants carrying AT70 and AT71 fragments in ‘CH8’ induced the expression of hybrid weakness. ‘LiyuB’ was used as the wild-type. Each line was represented by five independent transgenic plants. (C) Postulated model for *Hwc1*–*Hwc3* interaction leading to hybrid weakness in ‘CH7/8’ F_1_ hybrid, where X represents no transcription of the *Hwc3* gene and O represents transcription activation of the *Hwc3* gene due to the inserted promoter and transcription activator *Hwc1* in the ‘CH8’ genome. (This figure is available in color at *JXB* online.)

In order to verify that the gene at the *Hw*c*3* locus is the hybrid weakness gene, we performed a complementation test. A 2-kb fragment (designated AT70), which contained the retrotransposon together with the flanking LTR, and a 4-kb fragment (AT71), which contained the LTR retrotransposon, the promoter part and the gene *Os.89494* (Fig. 4A), were amplified from the ‘CH7’ genome. The fragments were cloned into a plant transformation binary vector, HPE203, and the constructs were used to transform ‘CH8’. Overall, 12 and 16 independent T_0_ transgenic plants were obtained from AT70 and AT71, respectively. We observed that 80% of the transgenic plants carrying AT71 exhibited the hybrid weakness phenotype, while plants carrying AT70 grew normally (Fig. 4B). This indicated that the hybrid weakness in the transgenic plants was associated with only AT71 and not with AT70, suggesting that the gene *Os.89494* was able to induce the weakness syndrome in ‘CH8’ only when the LTR retrotransposon was inserted, and that, without the LTR retrotransposon, *Os.89494* could not induce hybrid weakness, as depicted in the postulated model of hybrid weakness (Fig. 4C). Previously, Zhang (2012) had detected that the ‘CH8’ genome carried the rare hybrid weakness gene *Hwc1*. As the parental line carrying only *Hwc1* or *Hwc3* could grow and develop normally, it is suggested that the hybrid weakness observed in the F_1_ hybrids derived from ‘CH7/CH8’ resulted from the complementary action of two genes as previously reported (Chen *et al.*, 2014). We further considered that the insertion of the LTR retrotransposon into the promoter part of the *Hwc3* gene was transcribed by the transcriptional regulator, *Hwc1*, present in the ‘CH8’ genome. Although further experiments are needed to elucidate how genomic DNA from ‘CH8’ (*Hwc1*) induces the expression of *Hwc*3, our results preliminarily demonstrate that hybrid weakness in intrasubspecific *japonica* hybrids may be associated with a two-locus interaction. As the candidate *Hwc3* gene is generally not expressed under normal conditions, we speculate that the activation and expression of this gene are responsible for the hybrid weakness phenotype.

### The candidate gene of *Hwc3* encodes a leucine-rich repeat protein

*Hwc3* (*Os.89494*, *AK288361*) is 1341 bp. The candidate gene encodes a protein of 446 residues with 10 leucine-rich conserved domains (Supplementary Fig. S3). Apart from the leucine-rich repeats (LRR), no other functional domain was found in the gene. Protein homology analysis search by BLASTp showed that the *Hwc3* protein shared high sequence similarity with *XA1*, the bacterial rice blast*-*resistance protein from *O. sativa* (Supplementary Fig. S4). Analysis of the promoter region via Plant CARE software indicated the presence of eight *cis*-acting regulatory elements related to various physiological and metabolic processes. These include Skn-1, meristem specific activation, TATA box, and CAAT-box (Supplementary Table S5).

### Differential gene expression related to hybrid weakness

To obtain a global picture of gene expression changes that occurred in the F_1_ hybrids exhibiting the characteristic hybrid weakness phenotype, we performed two independent microarray analysis. Three biological replicates were used for each sample. These Affymetrix GeneChip arrays target 44 104 genes in the material studied. Putatively, differentially expressed genes involved in the hybrid weakness phenotype were identified by the following selection criteria: (i) statistically significant differences in gene expression were detected in both datasets; and (ii) the average of the fold-ratio change in expression level was greater than 2.0 or less than −2.0. The number of significantly differentially expressed genes was 443. Comparing the expression of these genes, our dataset indicated that transcript levels of 184 genes exhibited differential expression in the ‘CH7/CH8’ hybrid compared with either parent. Only those genes whose expression level showed a fold-ratio greater than +3.0 or less than −3.0 are listed in Table 1. Supplementary Fig. S5 shows a scatter plot graph and a heat map of expression-distribution patterns of differentially expressed genes after microarray hybridization with labeled cDNA probes obtained from mRNA of the studied materials. With the information from NCBI and Uniport databases, we assigned putative functions to the regulated genes and grouped them into functional categories depending on their biological function. Genes whose products had no known putative function or whose function was not very clear were grouped as unclassified genes. Results indicated that genes with altered expression were involved in various important cellular processes including metabolism, growth and development, signal transduction, and defense-related functions. Moreover, we noticed that among those differentially expressed genes, genes encoding metabolic pathways were over-represented. Contrary to previous studies (Bomblies and Weigel, 2007; Chen *et al.*, 2014), immune-related genes were not found to be differentially expressed, indicating that the hybrid weakness reported in this study was not related to autoimmunity. Differentially expressed genes involved in photosynthesis showed a high percentage of up-regulated expression, while differentially expressed genes involved in protein modifications and secondary metabolism exhibited a high proportion of down-regulated expression.

**Table 1.** List of differentially expressed genes in ‘CH7/8’ hybrid weakness F_1_ progeny

Annotation	Fold-change	Seq name	Unigene
Down-regulated genes			
Metabolism			
Thiosulfate sulfurtransferase	−3.0	chr6	*Os.10162*
Tubulin alpha-4A chain	−3.3	chr12	*Os.38240*
GDSL esterase/lipase	−3.5	chr5	*Os.7727*
Tryptophan N-hydroxylase 1	−4.9	chr4	*Os.18266*
Beta-glucosidase 16	−5.1	chr9	*Os.81365*
Phosphoribosylanthranilate transferase	−10.5	chr9	*Os.26406*
Cell growth/ development and other cellular processes			
Floral organ regulator 1 (FOR1)	−3.7	chr7	*Os.10733*
Cellular transportation/transportation			
Zinc transporter 5	−5.3	chr5	*Os.70563*
Phospholipid transfer protein precursor	−3.3	chr11	*Os.37890*
Cell defense and other cellular responses			
RING-H2 finger protein ATL80	−3.0	chr9	*Os.87833*
Secondary metabolites			
(−)-Germacrene D synthase-like	−5.3	chr4	*Os.11728*
*O*-Methyltransferase ZRP4-like	−6.1	chr9	*Os.53458*
Cell homeostasis			
Putative glutaredoxin-C12	−6.9	chr11	*Os.51468*
Unclassified or unknown proteins			
Unknown	−3.7	chr6	*XM_015787066.1*
Unknown	−5.7	chr4	*Os.87151*
Up-regulated genes			
Photosynthesis			
Cytochrome P450, family 78, subfamily A, polypeptide 7	19.7	chr10	*Os.46789*
Chlorophyll *a–b* binding protein 4	4.0	chr8	*Os.7890*
Cytosolic glutamine synthetase	7.6	chr3	*Os.12728*
Cell defense/rescue/responses			
Glutathione *S*-transferase	3.1	chr10	*Os.21842*
Cellular transportation/translocation			
Mitochondrial carnitine/acylcarnitine carrier-like protein	3.1	chr10	*Os.62649*
Translocon at the inner envelope membrane of chloroplasts 110	3.4	chr10	*Os.88107*
Transcription factor			
F-box protein PP2-B10-like	3.3	chr2	*Os.53262*
Unclassified or unknown proteins			
Unknown	3.8	chr12	*XM_015763550.1*
Unknown	3.1	chr9	*Os.55306*

Only those genes which show a fold-ratio greater than +3.0 or less than −3.0 are listed. The annotation identity is from BLAST.

## Discussion

### Genomic variations and intrasubspecific hybrid weakness in *japonica* rice

It is well known that the accumulation of genetic differences ultimately leads to reproductive isolation in plants (Widmer *et al.*, 2009; Baack *et al.*, 2015). As rice is a self-pollinated species, it is prone to accumulating mutations that can lead to genetic variations (Wright *et al.*, 2013). In rice, almost all kinds of reproductive isolation mechanisms reported so far in plants have been observed (Nadir *et al.*, 2018). Hybrid weakness is a common type of reproductive isolation mechanism that is frequently observed in hybrids between wild and cultivated rice or between *indica* and *japonica* rice (Ichitani *et al.* 2007, 2011; Kuboyama *et al.* 2009; Chen *et al.* 2013, 2014). Strong selection during the domestication process, mutation accumulation and adaptation to different environments are the key factors leading to the development of genetic differentiation and subsequently leading to hybrid incompatibilities between rice populations (Nadir *et al.*, 2018).

Some recent studies, including the current one, have reported that, in addition to interspecific and intersubspecific hybridization in rice, reproductive barriers in the form of hybrid weakness also exist between different lines of the same subspecies, *indica* or *japonica* (Zhang, 2012; Fu *et al.*, 2013). However, the exact understanding of the intrasubspecific or intervarietal hybrid weakness is still not very clear. In this study, the F_1_ generation clearly exhibited the hybrid weakness phenotype with its characteristic chlorotic phenotype, short stature, aberrant root formation, and markedly decreased tiller number (Figs 1, 2). Consistent with previous studies (Amemiya and Akemine, 1963; Chen *et al.*, 2013), the hybrid weakness symptoms became apparent at the seedling stage and became more obvious at tillering, indicating that hybrid weakness is expressed during plant development. Our results indicated abundant polymorphic sites (Supplementary Fig. S2; Supplementary Table S4) among the three *japonica* rice varieties, in the form of SNPs and InDels (Supplementary Fig. S1). We found that there was an uneven distribution of SNPs and InDels on the chromosomes. The DNA polymorphisms were mainly distributed in the intergenic, upstream, and downstream regions of genes. Fewer variants, resulting from InDels and SNPs, could be seen in protein-coding regions, splice junctions, and UTR regions. Non-coding regions that are highly conserved between species contain significantly fewer variants than other non-coding regions (Li *et al.*, 2015). These regions of the genes are mainly involved in the regulation of gene expression, so these changes might be involved in changes in gene expression. Similar results were previously reported for significant genetic polymorphism in the genomes of *indica* rice varieties RGD-7S and Taifeng B, whose F_1_ progeny exhibited the hybrid weakness phenotype (Fu *et al.*, 2016). Overall, these results indicated that the genetic backgrounds of these *japonica* rice varieties were very different and that they might have originated and evolved under very different conditions. This genome-level polymorphism might not complement one another when the two genomes are hybridized together, which probably leads to the defective development observed in the F_1_ progeny. The genome-wide polymorphism results obtained in this study will help further in designing InDel markers for further map-based cloning of hybrid weakness genes.

### Intrasubspecific hybrid weakness likely arose via an LTR retrotransposon

The results of the present study suggest that a *cis*-activation is caused by the insertion of an LTR retrotransposon in the promoter region of the *Hwc3* gene in ‘CH7’, which seems to enhance its expression in the F_1_ hybrid (Figs 3D, 4C). This was further supported by the genetic complementation tests, which showed that a 4-kb sequence of ‘CH7’, including the inserted LTR retrotransposon, the promoter, and the coding sequence of the *Hwc3* gene, could induce hybrid weakness in transgenic ‘CH8’ plants (Fig. 4B). The well-known explanation of the genetic basis of the evolution of hybrid incompatibilities comes from the Bateson–Dobzhansky–Muller (BDM) model, which states that hybrid incompatibility is due to negative interactions between at least two genetic loci (Johnson, 2008). However, many cases of hybrid incompatibility have been identified that showed other genetic basis for the evolution of hybrid incompatibilities, including chromosomal inversions, gene duplications, and gene transpositions. LTR retrotransposons are the most abundant class of transposons in plants (Wessler, 2006). Barbara McClintock noted that the activity of transposons can cause restructuring of the genome of hybrids. The involvement of a transposon or retrotransposon in inducing hybrid incompatibility has been previously reported in Arabidopsis and *Drosophila* (Blumenstiel and Hartl, 2005; Masly *et al.*, 2006; Josefsson *et al.*, 2006), although the transposon’s effects on hybrid fitness were not clear (Maheshwari and Barbash, 2011). Our study demonstrates that hybrid weakness in the present case is caused by a promoter on the LTR retrotransposon inducing gene expression at the *Hwc3* locus. The candidate *Hwc3* gene encoded an LRR protein (Supplementary Fig. S3), a type of protein that is mainly involved in protein–protein interactions. The *Hwc3* gene was found not to be expressed under normal conditions and the plants carrying this gene grew normally, which suggests that the gene has no apparent function and is a silenced gene. However, upon hybridization of ‘CH7’ with ‘CH8’, transcriptional activation occurred and the gene started to be expressed in the hybrid background, a finding that suggests that the ‘CH8’ genome contains some transcriptional factor that activates the gene expression in the F_1_ hybrid (Fig. 4C). Interactions between transcription factors and the promoter region of genes has been found to be an underlying mechanism of heterosis in rice F_1_ hybrids (Zhang *et al.*, 2008). Based on our current data, we hypothesize that the transcriptional activation of *Hwc3* by a transcriptional regulator, *Hwc1*, leads to hybrid weakness in *japonica* rice.

### Patterns and mechanisms of gene expression changes during hybrid weakness

To identify the candidate genes that play key roles in hybrid weakness in the intrasubspecific hybridization of *japonica* rice, we analysed differential gene expression profiling in the ‘CH7/8’ F_1_ hybrid at tillering by Affymetrix gene chip microarray. We found that the genes involved in the carbohydrate metabolism pathway had a higher proportion of down-regulated genes, while genes related to cellular defense, calcium signaling and secondary metabolism also tended to be down-regulated, as were genes related to protein biosynthesis and peptide transport (Supplementary Fig. S5; Table 1). Plant metabolism is highly coordinated with development as it influences the overall energy status of the plant (Creelman and Mullet, 1997; Ponnu *et al.*, 2011). Down-regulation of the genes associated with metabolism leads to drastic changes in the nutrient and energy balance of the plant and causes arrested growth and development (Ponnu *et al.*, 2011). The weakness phenotype of the F_1_ progeny observed in our study and the down-regulation of energy-metabolism-related genes might suggest that the observed phenotype could be due to the low energy status of the hybrids. Previously, carbohydrate metabolism-related genes were found to be up-regulated in heterotic rice hybrids (Zhang *et al.*, 2008; Wei *et al.*, 2009). Based on our data, we suggest that the weakness-associated phenotypic changes may result from quantitative differences in gene expression. The differences in gene expression pattern and gene expression level observed in our study might represent intravarietal expression polymorphisms. To dissect the complex mechanisms underlying heterosis, several researchers have attempted to identify genes leading to heterosis when species from different genetic backgrounds are crossed together (Guo *et al.*, 2004; Huang *et al.*, 2006; Zhu *et al.*, 2011). Changes in gene expression have been documented in hybrids of Arabidopsis (Andorf *et al.*, 2010; Fujimoto *et al.*, 2012; Shen *et al.*, 2012), *O. sativa* (Wei *et al.*, 2009; He *et al.*, 2010; Song *et al.*, 2010), *Zea mays* (Guo *et al.*, 2004; Stupar and Springer, 2006; Swanson-Wagner *et al.*, 2006; Lai *et al.*, 2010), and *Triticum* (Wang *et al.*, 2006). However, differential gene expression in relation to hybrid weakness had not been explored until this current study. The present study identified differentially expressed genes that represent a wide range of molecular functions. This will help to identify candidate hybrid weakness genes and to study their molecular mechanisms. As LRR-containing proteins are mainly involved in protein–protein interactions that regulate a variety of cellular functions, including cell cycle progression, signal transduction, and metabolic pathways, our results suggest that the expression of *Hwc3* might regulate the transcriptional functions of other proteins encoded by the differentially expressed genes in the ‘CH7/CH8’ F_1_ hybrid.

## Supplementary data

Supplementary data are available at *JXB* online.

Fig. S1. Proportions of InDels and SNPs in different gene regions of the *japonica* rice varieties ‘CH7’, ‘CH8’, and ‘CH9’.

Fig. S2. Sequence analysis at the *Hwc3* locus in ‘Nipponbare’, ‘CH7’, and ‘CH8’.

Fig. S3. The protein sequence of the candidate *Hwc3* gene product and its phylogeny.

Fig. S4. Protein homology of the *Hwc3* and *XA1* genes using the NCBI database.

Fig. S5. The differential gene expression patterns related to hybrid weakness.

Table S1. Primer sequences used in this study.

Table S2. Coverage of the reads mapping to the ‘Nipponbare’ reference genome from re-sequencing of the *japonica* rice varieties ‘CH7’, ‘CH8’, ‘CH9’, and ‘LiyuB’.

Table S3. Annotation of InDels identified in ‘CH7’, ‘CH8’ and ‘CH9’ and ‘LiyuB’ in comparison with ‘Nipponbare’.

Table S4. Annotation of SNPs identified in ‘CH7’, ‘CH8’ and ‘CH9’ and ‘LiyuB’ in comparison with ‘Nipponbare’.

Table S5. Bioinformatics analysis of the promoter structure of *Hwc*3.

Supplementary Figures S1-S5

Supplementary Tables S1-S5

## References

[CIT0001] Andorf S, Selbig J, Altmann T, Poos K, Witucka-Wall H, Repsilber D 2010 Enriched partial correlations in genome-wide gene expression profiles of hybrids (*A. thaliana*): a systems biological approach towards the molecular basis of heterosis. Theoretical and Applied Genetics 120, 249–259. 19921139 10.1007/s00122-009-1214-z

[CIT0002] Amemiya A, Akemine H 1963 Biochemical genetic studies on the root growth inhibiting complementary lethals in rice plant (Studies on the embryo culture in rice plant. 3). Bulletin of the National Institute of Agricultural Sciences (Japan) D10, 139–226 [in Japanese].

[CIT0003] Baack E, Melo MC, Rieseberg LH, Barrientos DO 2015 The origins of reproductive isolation in plants. New Phytologist 207, 968–984. 25944305 10.1111/nph.13424

[CIT0004] Bomblies K, Weigel D 2007 Hybrid necrosis: autoimmunity as a potential gene-flow barrier in plant species. Nature Reviews Genetics 8, 382–393. 10.1038/nrg2082 17404584

[CIT0005] Blumenstiel JP, Hartl DL 2005 Evidence for maternally transmitted small interfering RNA in the repression of transposition in *Drosophila virilis*. Proceedings of the National Academy of Sciences, USA 102, 15965–15970. 10.1073/pnas.0508192102 PMC1276106 16247000

[CIT0006] Chambeyron S, Popkova A, Payen-Groschêne G, Brun C, Laouini D, Pelisson A, Bucheton A 2008 piRNA-mediated nuclear accumulation of retrotransposon transcripts in the *Drosophila* female germ line. Proceedings of the National Academy of Sciences, USA 105, 14964–14969. 10.1073/pnas.0805943105 PMC2567476 18809914

[CIT0007] Chen C, Chen H, Lin YS, et al 2014 A two-locus interaction causes interspecific hybrid weakness in rice. Nature Communications 5, 3357. 10.1038/ncomms4357 PMC3948059 24556665

[CIT0008] Chen C, Chen H, Shan JX, Zhu MZ, Shi M, Gao JP, Lin HX 2013 Genetic and physiological analysis of a novel type of interspecific hybrid weakness in rice. Molecular Plant 6, 716–728. 23220941 10.1093/mp/sss146

[CIT0009] Chu Y, Oka H 1972 Distribution and effects of genes causing F1 weakness in *Oryza breviligulata* and *O. glaberrima*. Genetics 70, 163–173. 17248554 10.1093/genetics/70.1.163 PMC1212718

[CIT0010] Cingolani P, Patel VM, Coon M, Nguyen TL, Ruden SJ, Lu X 2012 Using *Drosophila melanogaster* as a model for genotoxic chemical mutational studies with a new program, SnpSift. Frontiers in Genetics 3, 35. 22435069 10.3389/fgene.2012.00035 PMC3304048

[CIT0011] Coyne JA, Orr HA 2004 Speciation. Sunderland, MA, USA: Sinauer Associates, Inc.

[CIT0012] Creelman RA, Mullet JE 1997 Oligosaccharins, brassinolides, and jasmonates: nontraditional regulators of plant growth, development, and gene expression. The Plant Cell 9, 1211–1223. 9254935 10.1105/tpc.9.7.1211 PMC156992

[CIT0013] Doyle JJ, Doyle JL 1990 Isolation of plant DNA from fresh tissue. Focus 12, 13–15.

[CIT0014] Fu CY, Liu WG, Liu DL, Li JH, Zhu MS, Liao YL, Liu ZR, Zeng XQ, Wang F 2016 Genome-wide DNA polymorphism in the *indica* rice varieties RGD-7S and Taifeng B as revealed by whole genome re-sequencing. Genome 59, 197–207. 26926666 10.1139/gen-2015-0101

[CIT0015] Fu CY, Wang F, Sun BR, et al 2013 Genetic and cytological analysis of a novel type of low temperature-dependent intrasubspecific hybrid weakness in rice. PLoS ONE 8, e73886. 24023693 10.1371/journal.pone.0073886 PMC3758327

[CIT0016] Fujimoto R, Taylor JM, Shirasawa S, Peacock WJ, Dennis ES 2012 Heterosis of *Arabidopsis* hybrids between C24 and Col is associated with increased photosynthesis capacity. Proceedings of the National Academy of Sciences, USA 109, 7109–7114. 10.1073/pnas.1204464109 PMC3344962 22493265

[CIT0017] Guo M, Rupe MA, Zinselmeier C, Habben J, Bowen BA, Smith OS 2004 Allelic variation of gene expression in maize hybrids. The Plant Cell 16, 1707–1716. 15194819 10.1105/tpc.022087 PMC514155

[CIT0018] Hannah MA, Krämer KM, Geffroy V, et al 2007 Hybrid weakness controlled by the dosage-dependent lethal (DL) gene system in common bean (*Phaseolus vulgaris*) is caused by a shoot-derived inhibitory signal leading to salicylic acid-associated root death. New Phytologist 176, 537–549. 17850251 10.1111/j.1469-8137.2007.02215.x

[CIT0019] Hatano H, Mizuno N, Matsuda R, Shitsukawa N, Park P, Takumi S 2012 Dysfunction of mitotic cell division at shoot apices triggered severe growth abortion in interspecific hybrids between tetraploid wheat and *Aegilops tauschii*. New Phytologist 194, 1143–1154. 22436033 10.1111/j.1469-8137.2012.04125.x

[CIT0020] He G, Zhu X, Elling AA, et al 2010 Global epigenetic and transcriptional trends among two rice subspecies and their reciprocal hybrids. The Plant Cell 22, 17–33. 20086188 10.1105/tpc.109.072041 PMC2828707

[CIT0021] Huang Y, Zhang L, Zhang J, Yuan D, Xu C, Li X, Zhou D, Wang S, Zhang Q 2006 Heterosis and polymorphisms of gene expression in an elite rice hybrid as revealed by a microarray analysis of 9198 unique ESTs. Plant Molecular Biology 62, 579–591. 16941221 10.1007/s11103-006-9040-z

[CIT0022] Ichitani K, Fukuta Y, Taura S, Sato M 2001 Chromosomal location of *Hwc2*, one of the complementary hybrid weakness genes, in rice. Plant Breeding 120, 523–525.

[CIT0023] Ichitani K, Namigoshi K, Sato M, Taura S, Aoki M, Matsumoto Y, Saitou T, Marubashi W, Kuboyama T 2007 Fine mapping and allelic dosage effect of *Hwc1*, a complementary hybrid weakness gene in rice. Theoretical and Applied Genetics 114, 1407–1415. 17375279 10.1007/s00122-007-0526-0

[CIT0024] Ichitani K, Taura S, Tezuka T, Okiyama Y, Kuboyama T 2011 Chromosomal location of *HWA1* and *HWA2*, complementary hybrid weakness genes in rice. Rice 4, 29–38.

[CIT0025] Josefsson C, Dilkes B, Comai L 2006 Parent-dependent loss of gene silencing during interspecies hybridization. Current Biology 16, 1322–1328. 16824920 10.1016/j.cub.2006.05.045

[CIT0026] Johnson NA 2008 Hybrid incompatibility and speciation. Nature Education 1, 20.

[CIT0027] Koide Y, Onishi K, Kanazawa A, Sano Y 2008 Genetics of speciation in rice. In: Hirano H-Y, Sano Y, Hirai A, Sasaki T, eds. Rice biology in the genomics era. Berlin: Springer.

[CIT0028] Kuboyama T, Saito T, Matsumoto T, Wu J, Kanamori H, Taura S, Sato M, Marubashi W, Ichitani K 2009 Fine mapping of *HWC2*, a complementary hybrid weakness gene, and haplotype analysis around the locus in rice. Rice 2, 93–103.

[CIT0029] Kirkpatrick M, Barton N 2006 Chromosome inversions, local adaptation and speciation. Genetics 173, 419–434. 16204214 10.1534/genetics.105.047985 PMC1461441

[CIT0030] Lai J, Li R, Xu X, et al 2010 Genome-wide patterns of genetic variation among elite maize inbred lines. Nature Genetics 42, 1027–1030. 20972441 10.1038/ng.684

[CIT0031] Li D, Zeng R, Li Y, Zhao M, Chao J, Li Y, Wang K, Zhu L, Tian WM, Liang C 2015 Gene expression analysis and SNP/InDel discovery to investigate yield heterosis of two rubber tree F1 hybrids. Scientific Reports 6, 24984. 10.1038/srep24984 PMC4842955 27108962

[CIT0032] Li H, Durbin R 2009 Fast and accurate short read alignment with Burrows-Wheeler transform. Bioinformatics 25, 1754–1760. 19451168 10.1093/bioinformatics/btp324 PMC2705234

[CIT0033] Lynch M, Force AG 2000 The origin of interspecific genomic incompatibility via gene duplication. The American Naturalist 156, 590–605. 10.1086/316992 29592543

[CIT0034] Maheshwari S, Barbash DA 2011 The genetics of hybrid incompatibilities. Annual Review of Genetics 45, 331–355. 10.1146/annurev-genet-110410-132514 21910629

[CIT0035] Masly JP, Jones CD, Noor MA, Locke J, Orr HA 2006 Gene transposition as a cause of hybrid sterility in *Drosophila*. Science 313, 1448–1450. 16960009 10.1126/science.1128721

[CIT0036] Nadir S, Khan S, Zhu Q, Henry D, Wei L, Lee DS, Chen LJ 2018 An overview on reproductive isolation in *Oryza sativa* complex. AoB Plants 10, ply060. 30538811 10.1093/aobpla/ply060 PMC6280023

[CIT0037] Oka HI 1957 Phylogenetic differentiation of cultivated rice. XV: Complementary lethal genes in rice. Japanese Journal of Genetics 32, 83–87.

[CIT0038] Okuno K, Fukuoka S 1999 Distribution and RFLP mapping of complementary genes causing hybrid breakdown in Asian cultivated rice, *Oryza sativa* L. JARQ 33, 1–6.

[CIT0039] Ponnu J, Wahl V, Schmid M 2011 Trehalose-6-phosphate: connecting plant metabolism and development. Frontiers in Plant Science 2, 70. 22639606 10.3389/fpls.2011.00070 PMC3355582

[CIT0040] Rebollo R, Romanish MT, Mager DL 2012 Transposable elements: an abundant and natural source of regulatory sequences for host genes. Annual Review of Genetics 46, 21–42. 10.1146/annurev-genet-110711-155621 22905872

[CIT0041] Rieseberg LH, Willis JH 2007 Plant speciation. Science 317, 910–914. 17702935 10.1126/science.1137729 PMC2442920

[CIT0042] Song GS, Zhai HL, Peng YG, et al 2010 Comparative transcriptional profiling and preliminary study on heterosis mechanism of super-hybrid rice. Molecular Plant 3, 1012–1025. 20729474 10.1093/mp/ssq046 PMC2993235

[CIT0043] Shen H, He H, Li J, et al 2012 Genome-wide analysis of DNA methylation and gene expression changes in two *Arabidopsis* ecotypes and their reciprocal hybrids. The Plant Cell 24, 875–892. 22438023 10.1105/tpc.111.094870 PMC3336129

[CIT0044] Stupar RM, Springer NM 2006 *Cis*-transcriptional variation in maize inbred lines B73 and Mo17 leads to additive expression patterns in the F_1_ hybrid. Genetics 173, 2199–2210. 16702414 10.1534/genetics.106.060699 PMC1569691

[CIT0045] Swanson-Wagner RA, Jia Y, DeCook R, Borsuk LA, Nettleton D, Schnable PS 2006 All possible modes of gene action are observed in a global comparison of gene expression in a maize F_1_ hybrid and its inbred parents. Proceedings of the National Academy of Science, USA 103, 6805–6810. 10.1073/pnas.0510430103 PMC1447595 16641103

[CIT0046] Wang J, Tian L, Lee HS, et al 2006 Genomewide nonadditive gene regulation in Arabidopsis allotetraploids. Genetics 172, 507–517. 16172500 10.1534/genetics.105.047894 PMC1456178

[CIT0047] Wessler SR 2006 Transposable elements and the evolution of eukaryotic genomes. Proceedings of the National Academy of Sciences, USA 103, 17600–17601. 10.1073/pnas.0607612103 PMC1693792 17101965

[CIT0048] Widmer A, Lexer C, Cozzolino S 2009 Evolution of reproductive isolation in plants. Heredity 102, 31–38. 18648386 10.1038/hdy.2008.69

[CIT0049] Wright SI, Kalisz S, Slotte T 2013 Evolutionary consequences of self-fertilization in plants. Proceedings of the Royal Society. Series B, Biological Sciences 280, 20130–20133. 10.1098/rspb.2013.0133 PMC3652455 23595268

[CIT0050] Wei G, Tao Y, Liu GZ, et al 2009 A transcriptomic analysis of super hybrid rice LYP9 and its parents. Proceedings of the National Academy of Sciences, USA 106, 7695–7701. 10.1073/pnas.0902340106 PMC2683082 19372371

[CIT0051] Wei ZF 2013 Cloning and variation analysis of the candidate genes conferring hybrid weakness in japonica rice (Oryza sativa L.). MS Thesis. Yunnan Agriculture University, Kunming, China.

[CIT0052] Yang F 2005 Genetic characterization and molecular markers of hybrid weakness in rice. MS Thesis. Yunnan Agriculture University, Kunming, China.

[CIT0053] Yu X, Zhao Z, Zheng X, et al 2018 A selfish genetic element confers non-Mendelian inheritance in rice. Science 360, 1130–1132. 29880691 10.1126/science.aar4279

[CIT0054] Zhang H 2012 Phenotypic characterization and molecular bases of hybrid weakness in japonica rice (Oryza sativa L.). PhD Thesis. Yunnan Agriculture University, Kunming, China.

[CIT0055] Zhang HY, He H, Chen LB, et al 2008 A genome-wide transcription analysis reveals a close correlation of promoter INDEL polymorphism and heterotic gene expression in rice hybrids. Molecular Plant 1, 720–731. 19825576 10.1093/mp/ssn022

[CIT0056] Zhu X, Ainijiang, Zhang Y, Guo W, Zhang TZ 2011 Relationships between differential gene expression and heterosis in cotton hybrids developed from the foundation parent CRI-12 and its pedigree-derived lines. Plant Science 180, 221–227. 21421364 10.1016/j.plantsci.2010.08.011

