# Supplementary material for: A novel discovery of a long terminal repeat retrotransposon-induced hybrid weakness in rice
Source: J Exp Bot. 2018 Dec 20;70(4):1197–207. doi: 10.1093/jxb/ery442 (PMC6382335; doi:10.1093/jxb/ery442)
Supplement: Supplementary Figures S1-S5 [file ery442_suppl_supplementary_figures_s1-s5.pdf]

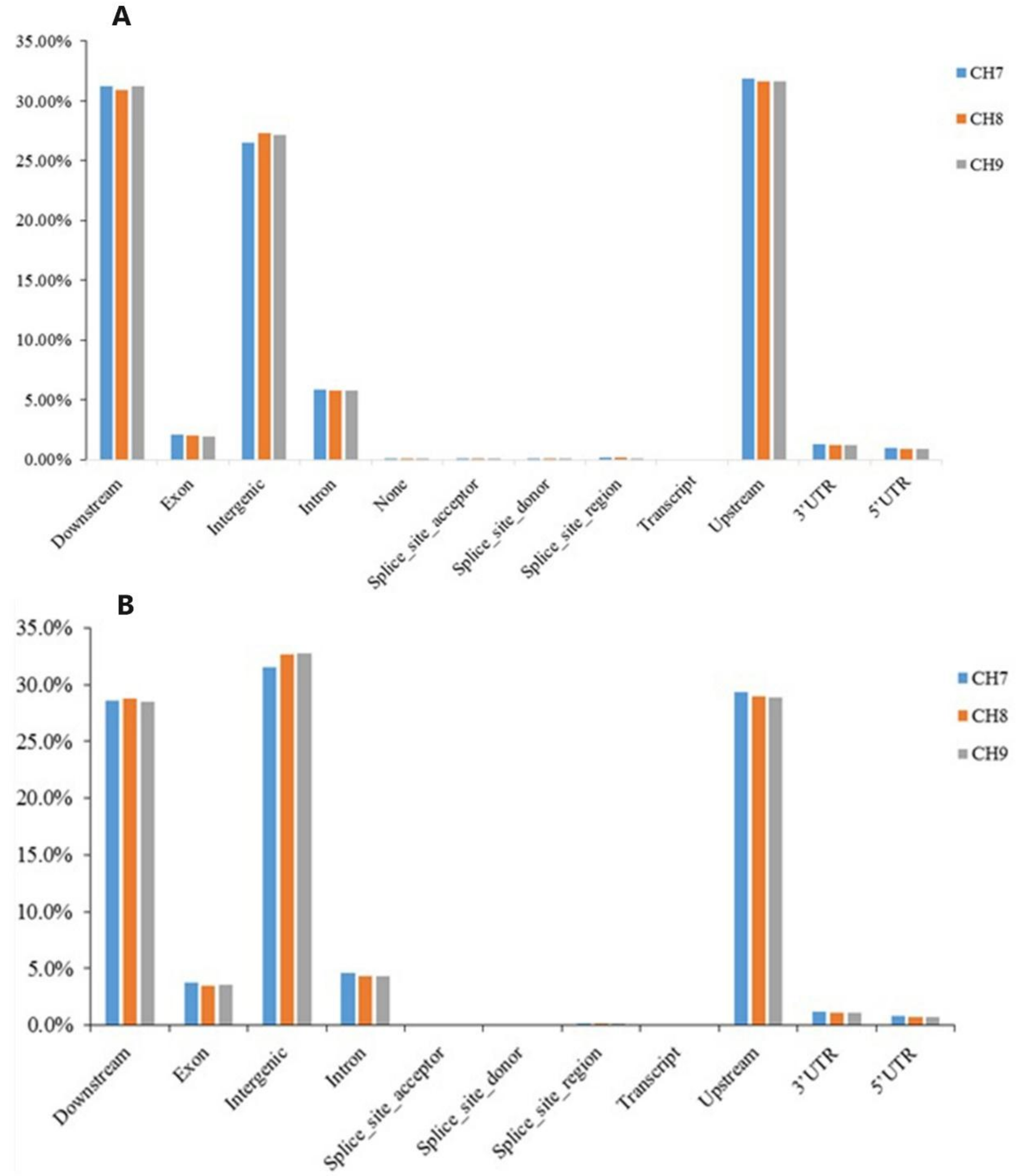

Fig. S1: Proportions of InDels (A) and SNPs (B) in different gene regions of the *japonica* rice varieties ‘CH7’, ‘CH8’ and ‘CH9’.

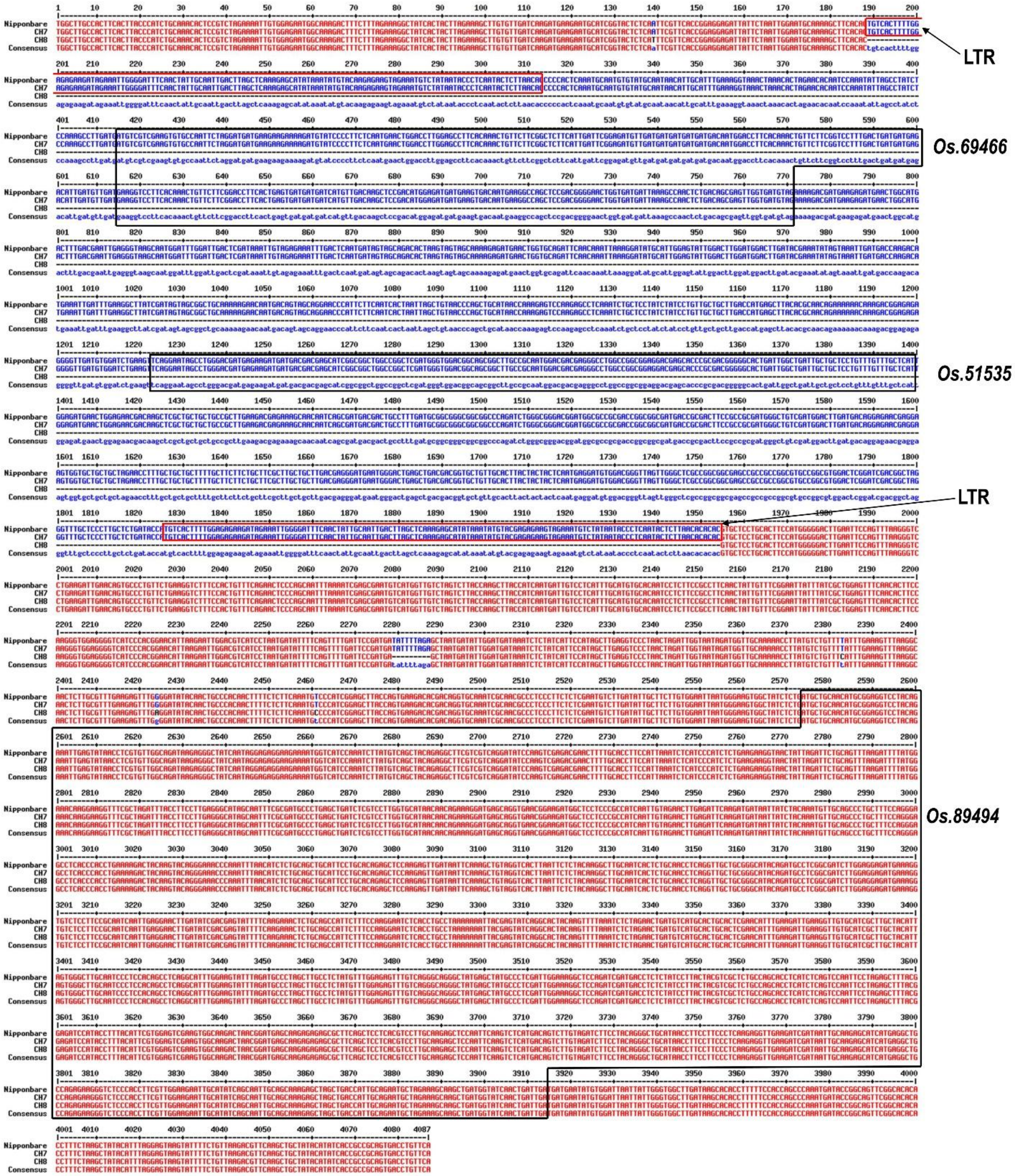

Fig. S2: Sequence analysis at *Hwc3* locus in 'Nipponbare', 'CH7' and 'CH8'.

A

MLQHAEVLQKLSITSCWQIRGLSIGEEENGHPNLMSATEASSSGYPSRDELLHLPLNLIPSLKKVTIRFCSLRF
YGNKEGFARFTFLEGIAISRCPELISSLVHNNRKDEQVNGRWLLPPSIVELEIQDDNYLQMLQPCFPGSLTHL
KRLQVQGNPNLTSLQLHSCTELQELIQSCRSLNSLQGLQSLCNLRLLRAYRCLGDLGGDERCLLPQSIEELDID
EYFQETLQPFFPRNLTCLKKLRVSGTTSFKSLELMSCTALEHLKIEGCASLATLVGLQSLHSLRHLEVFRCPSLPL
CLESLSGQGYELCPRLERLQIDDLSILTTSLCQHLISVQFLELYGDPYLYIRGVEVARLTDEQERALQLLTSLQEL
QFKSHDSLVDLPTGLHNLPSLKRLKIDNCKSIMRLPEKGLPPSLEELHISNCSKELADHCRMLESKLMVSTD

B

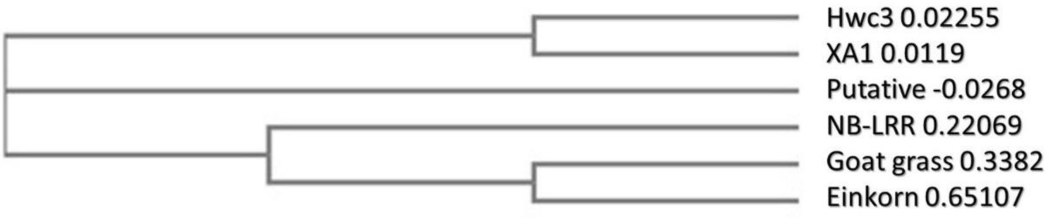

Fig. S3: The protein sequence of the candidate *Hwc3* gene product and its phylogeny.

(A) The protein encoded by *Hwc3*. Leucine-rich repeats are shown in colors. (B) Phylogenetic analysis of proteins encoded by *Hwc3* and other LRR-containing proteins.

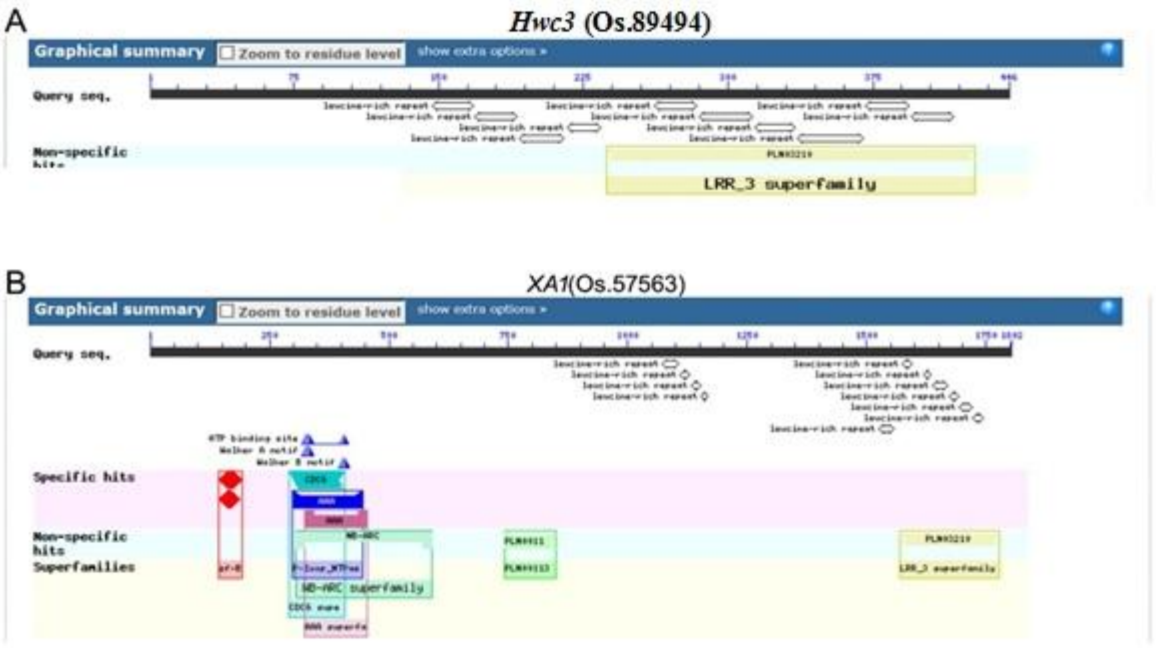

Fig. S4: Protein homology of *Hwc3* and *XA1* genes using the NCBI database

(A) The candidate gene *Hwc3* (*Os.89494)*. **(B)** The rice blast resistance gene *XA1*. The leucine-rich repeats can be seen in the figure.

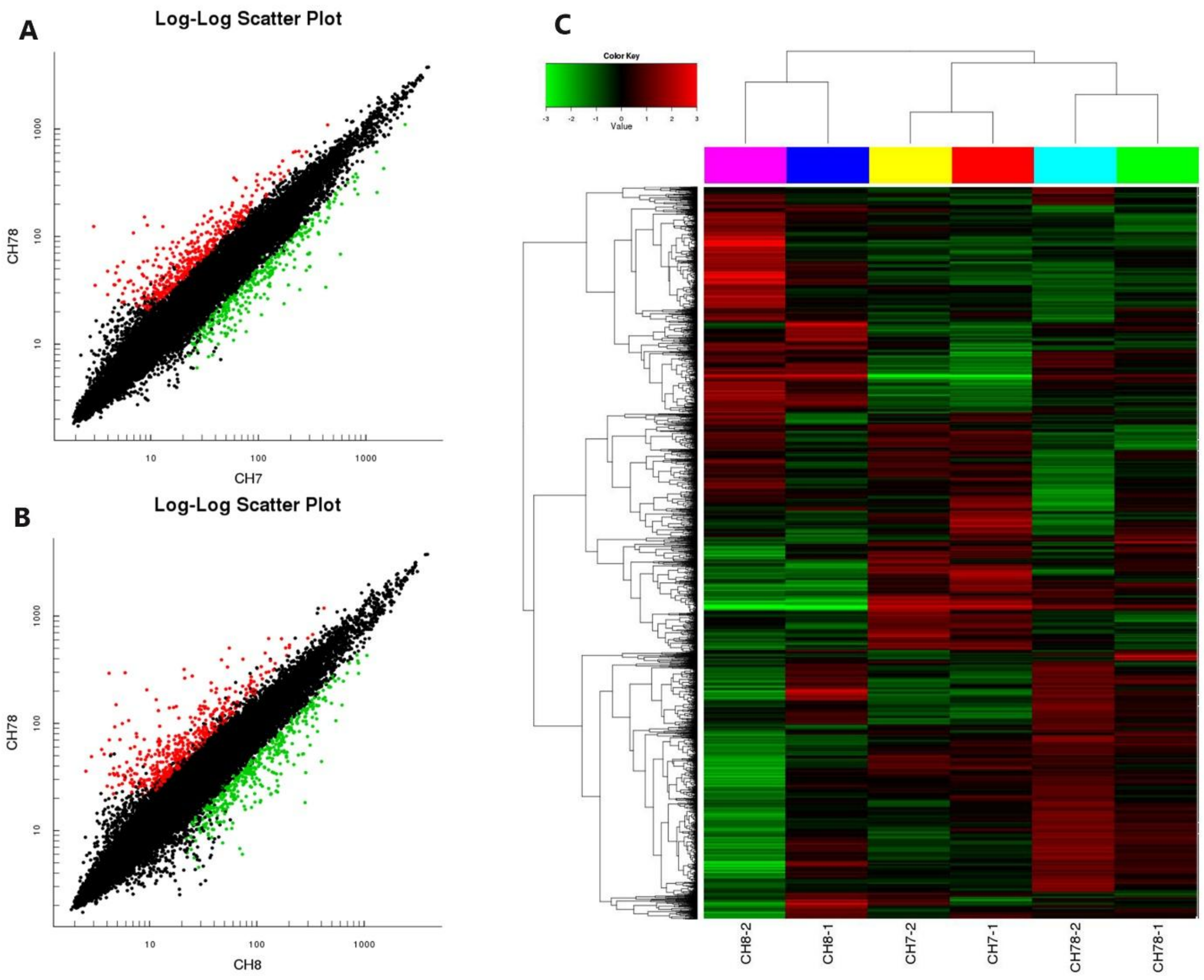

Fig. S5: The differential gene expression patterns related to hybrid weakness.

(A) Log-log scatter plots for gene expression comparison between 'CH7' and 'CH7/8'. **(B)** Log-log scatter plots for gene expression comparison between 'CH8' and 'CH7/8'. **(C)** Heat map of differential gene expression of 'CH7', 'CH8' and 'CH7/8' F_1 hybrid exhibiting hybrid weakness.
