# Supplementary material for: A novel discovery of a long terminal repeat retrotransposon-induced hybrid weakness in rice
Source: J Exp Bot. 2018 Dec 20;70(4):1197–207. doi: 10.1093/jxb/ery442 (PMC6382335; doi:10.1093/jxb/ery442)
Supplement: Supplementary Tables S1-S5 [file ery442_suppl_supplementary_tables_s1-s5.pdf]

Table S1: Primer sequences used in this study.

**Primer**	**Sequence**
Os89494-F	TGGGCAAATGGGGTCTCGTCT
Os89494-R	GCGGTTGTACCATCCGGTGA
Os51535-F	CAAACCCTAGCCGTCGATCCG
Os51535-R	ACCCAGCTGCATAACCAAAGAGT
Os69466-F	ACTATTGCAATTGACTTAGCTCAAAGA
Os69466-R	AGTTCATCTCTTCATCGTCTTTTCT
β-ACTIN 1D-F	GTTTGAGACCTTCAACACCCCT
β-ACTIN 1D-R	CTGGTCTTGGCAGTCTCCATTT
*Hwc3*a-F	CACTTACCCATCTGCAAACACT
*Hwc3*a-R	TAGACAACCATGACATTCGCTC
*Hwc3*b-F	TGCATCGGTACTCTCAATTCGT
*Hwc3*b-R	ACTGCGGCGGTGATATGTATAG

Table S2: Coverage of the reads mapping to the 'Nipponbare' reference genome from re-sequencing of the *japonica* rice varieties 'CH7', 'CH8', 'CH9' and 'LiyuB'.

**Sample**	**Raw reads numbers**	**Raw bases**	**Clean reads number**	**Clean bases**	**Clean rate (%)**	**Q20 (%)**	**Q30 (%)**
CH7	129994250	19444009425	123792816	18515955908	95.23	97.72	93.82
CH8	149107224	22254685078	142658002	21290400360	95.67	97.82	94.06
CH9	131824382	19711183775	124915310	18677257160	94.76	97.68	93.88
LiyuB	138104722	20625558732	131997874	19712395423	95.58	97.74	93.98

Table S3: Annotation of InDels that were identified in 'CH7', 'CH8', 'CH9' and 'LiyuB' in comparison to 'Nipponbare'.

	**CH7**	**CH8**	**CH9**	**LiyuB**
**Type**	**Count**	**Count**	**Count**	**Count**
Downstream	27026	47362	33205	28669
Exon	1846	2978	2161	1861
Intergenic	22917	41227	29306	24298
Intron	5102	8803	6230	5284
None	16	23	22	11
Splice_site_acceptor	15	28	18	15
Splice_site_donor	10	20	16	11
Splice_site_region	127	195	152	124
Transcript	-	3	2	-
Upstream	27567	47997	33995	28986
3'UTR	1134	1808	1294	1076
5'UTR	827	1317	946	847
**Total**	86587	151761	107347	91182

Table S4: Annotation of SNPs that were identified in ‘CH7’, ‘CH8’, ‘CH9’ and ‘LiyuB’ in comparison to ‘Nipponbare’.

	**CH7**	**CH8**	**CH9**	**LiyuB**
**Type**	**Count**	**Count**	**Count**	**Count**
Downstream	139445	251449	195190	136687
Exon	18500	30090	24641	17851
Intergenic	153747	285907	224598	151584
Intron	22491	37523	29491	20379
Splice_site_acceptor	23	33	23	24
Splice_site_donor	20	32	21	14
Splice_site_region	697	1098	889	669
Transcript	21	32	40	22
Upstream	143104	252924	197928	139025
3’UTR	5928	9355	7217	5376
5’UTR	3988	6224	5080	3678
**Total**	487964	874667	685118	475309

Table S5: Bioinformatics analysis of the promoter structure of *Hwc3*.

**Site Name**	**Sequence**	**Function**
Box 4	ATTAAT	part of a conserved DNA module involved in light responsiveness
CAAT-box	CAAAT<br>CAAT	common *cis*-acting element in promoter and enhancer regions
CAT-box	GCCACT	*cis*-acting regulatory element related to meristem expression
G-box	CACGAC	*cis*-acting regulatory element involved in light responsiveness
MBS	CAACTG	MYB binding site involved in drought-inducibility
Sp1	CC(G/A)CCC	light responsive element
TATA-box	TATA	core promoter element around -30 of transcription start
Unnamed_4	CTCC	Unknown
